# Association of time of breakfast and nighttime fasting duration with breast cancer risk in the multicase-control study in Spain

**DOI:** 10.3389/fnut.2022.941477

**Published:** 2022-08-11

**Authors:** Anna Palomar-Cros, Barbara N. Harding, Ana Espinosa, Kyriaki Papantoniou, Beatriz Pérez-Gómez, Kurt Straif, Eva Ardanaz, Tania Fernández Villa, Pilar Amiano, Inés Gómez-Acebo, Victor Moreno, Juan Alguacil, Guillermo Fernández-Tardón, Ana Molina-Barceló, Rafael Marcos-Gragera, Nuria Aragonés, Gemma Castaño-Vinyals, Marcela Guevara, Alba Marcos Delgado, Marina Pollán, Dora Romaguera, Manolis Kogevinas

**Affiliations:** ^1^Barcelona Institute for Global Health (ISGlobal), Barcelona, Spain; ^2^Department of Medicine and Life Sciences, Universitat Pompeu Fabra (UPF), Barcelona, Spain; ^3^Hospital del Mar Medical Research Institute (IMIM), Barcelona, Spain; ^4^Consortium for Biomedical Research in Epidemiology and Public Health (CIBERESP), Institute of Health Carlos III, Madrid, Spain; ^5^Department of Epidemiology, Centre of Public Health, Medical University of Vienna, Vienna, Austria; ^6^National Centre for Epidemiology, Carlos III Institute of Health, Madrid, Spain; ^7^Boston College, Chestnut Hill, MA, United States; ^8^Navarra Public Health Institute, Pamplona, Spain; ^9^Navarra Institute for Health Research (IdiSNA), Pamplona, Spain; ^10^Research Group on Gene-Environment Interactions and Health (GIIGAS), Institute of Biomedicine (IBIOMED), Universidad de León, León, Spain; ^11^Ministry of Health of the Basque Government, Sub-Directorate for Public Health and Addictions of Gipuzkoa, Gipuzkoa, Spain; ^12^Group of Epidemiology of Chronic and Communicable Diseases, Biodonostia Health Research Institute, San Sebastián, Gipuzkoa, Spain; ^13^Faculty of Medicine, University of Cantabria, Santander, Spain; ^14^Cancer Epidemiology Research Program, Instituto de Investigación Biomédica de Bellvitge (IDIBELL), Hospitalet de Llobregat, Barcelona, Spain; ^15^Catalan Institute of Oncology, Hospitalet de Llobregat, Barcelona, Spain; ^16^Department of Clinical Sciences, Faculty of Medicine, University of Barcelona, Barcelona, Spain; ^17^Centre for Health and Environmental Research, Huelva University, Huelva, Spain; ^18^Unit of Molecular Cancer Epidemiology, Department of Medicine, University Institute of Oncology of the Principality of Asturias (IOUPA), University of Oviedo, Oviedo, Spain; ^19^Health Research Institute of the Principality of Asturias (ISPA), Oviedo, Spain; ^20^Cancer and Public Health Area, Foundation for the Promotion of the Research in Healthcare and Biomedicine (FISABIO-Salud Pública), Valencia, Spain; ^21^Epidemiology Unit and Girona Cancer Registry, Oncology Coordination Plan, Department of Health, Autonomous Government of Catalonia, Catalan Institute of Oncology (ICO), Girona Biomedical Research Institute (IdiBGi), Girona, Spain; ^22^Epidemiology Section, Public Health Division, Department of Health of Madrid, Madrid, Spain; ^23^Health Research Institute of the Balearic Islands (IdISBa), Palma, Spain; ^24^Centro de Investigación Biomédica en Red de la Fisiopatología de la Obesidad y Nutrición (CIBEROBN), Madrid, Spain

**Keywords:** meal timing, circadian nutritional behaviors, nighttime fasting duration, breakfast, breast cancer risk, chrononutrition, circadian rhythms

## Abstract

Circadian nutritional behaviors, defined by the daily eating/fasting cycle, have been linked with breast cancer. This study aimed to further disentangle the association of nighttime fasting duration and time of breakfast with breast cancer risk. We analyzed data from 1,181 breast cancer cases and 1,326 population controls from the Spanish multicase-control study (MCC-Spain), 2008–2013. We collected circadian nutritional behaviors at mid-age *via* a telephonic interview. We applied logistic regression to estimate odds ratios (OR) and 95% confidence intervals (CIs) for the association of nighttime fasting duration and time of breakfast with breast cancer risk in all women and stratified by menopausal status. Models were adjusted for age, center, education, family history of breast cancer, age at menarche, number of children, breastfeeding, age at first child, body mass index (BMI), contraceptive use, and hormonal replacement therapy (HRT). A later time of breakfast was associated with a non-significant increased risk of breast cancer (OR = 1.05, 95% CI: 0.95–1.16, per hour increase). This association was stronger among premenopausal women, among whom each hour later, the time of breakfast was associated with an 18% increase in breast cancer risk (OR = 1.18, 95% CI: 1.01–1.40). The association was not observed in postmenopausal women. We did not observe an association between nighttime fasting duration and breast cancer risk after adjusting for the time of breakfast. In this study, late breakfast was associated with increased breast cancer risk, especially among premenopausal women, compared with early breakfast. Aside from nutritional quality, circadian nutritional behaviors should be further studied in relation to cancer.

## Introduction

Female breast cancer was the most commonly diagnosed cancer and the fifth leading cause of cancer-related mortality worldwide in 2020 (1). It was first proposed during the 1970s that the disruption of circadian rhythms could influence breast cancer risk (2). On the basis of a growing body of evidence, the International Agency for Research on Cancer (IARC) classified circadian rhythm disruption, resulting from night shift work, as probably carcinogenic for cancer of the breast, prostate, and colon (3, 4). Circadian rhythms regulate multiple physiological activities including hormonal secretion, immune regulation, and cellular cycle (5). Several external factors or *zeitgebers* can synchronize the circadian rhythms including the daily light-dark and feeding-fasting cycles (5).

The emerging field of chrononutrition studies the relationship between the timing of nutritional behaviors, circadian rhythms, and health (6–8). Some studies have shown that circadian nutritional behaviors, or meal timings, may be associated with breast cancer risk and progression (9–11). A prolonged nightly period of fasting has been associated with reduced systemic inflammation (12), a putative risk factor for breast cancer. Fasting for less than 13 h overnight has been associated with increased odds of breast cancer recurrence compared with a longer nightly fasting period (11). Contrarily, a study from the French cohort NutriNet-Santé showed no association between the length of the nightly fasting period and the risk of breast cancer (10).

The nightly fasting interval can be elongated either by having an early dinner or by having a late breakfast. Results from the multicase-control study (MCC-Spain) and the NutriNet-Santé cohort showed that having an early dinner was associated with a reduced risk of breast cancer compared with a late dinner (9, 10). In contrast, previous studies indicate that skipping breakfast, or delaying the first meal, can lead to metabolic and inflammatory deregulation (13–17). It has also been inconsistently linked with weight gain (14, 18, 19). The omission of breakfast has also been associated with increased cancer-related and all-cause mortality (20). Finally, data from the NutriNet-Santé cohort shows a non-significant association between a later breakfast and a higher risk of breast cancer (10). These analyses were not stratified by menopausal status.

Some cross-sectional studies have suggested an association between a prolonged nightly fasting period and a reduction in potential breast cancer risk factors (12, 21). However, the evidence is scarce and inconclusive. Moreover, the association with the time of breakfast and with consideration of menopausal status remains unclear. This analysis builds on previous results within the MCC-Spain study in relation to the time of dinner and the time interval between dinner and sleep (9) to build a more integrated understanding of the circadian nutritional behaviors as a whole. This study investigates whether circadian nutritional behaviors, specifically nighttime fasting duration and time of breakfast, are associated with breast cancer risk.

## Materials and methods

### Study design and population

The multicase-control MCC-Spain study^1^ is a large population-based case-control study of 5 common tumors, which was conducted in Spain between 2008 and 2013 (22, 23). Histologically confirmed cancer cases were recruited from 23 collaborating hospitals in 12 Spanish provinces. Simultaneously, controls were randomly selected from the primary healthcare centers located within the catchment area and were frequency-matched to cases by age, sex, and region. All participants were aged 20–85 years and had resided in the catchment area for 6 months or more prior to recruitment. For each of the included centers, the ethics committees reviewed and approved the study protocol. Before being included in this study, participants signed an informed consent form (22).

**FIGURE 1:**
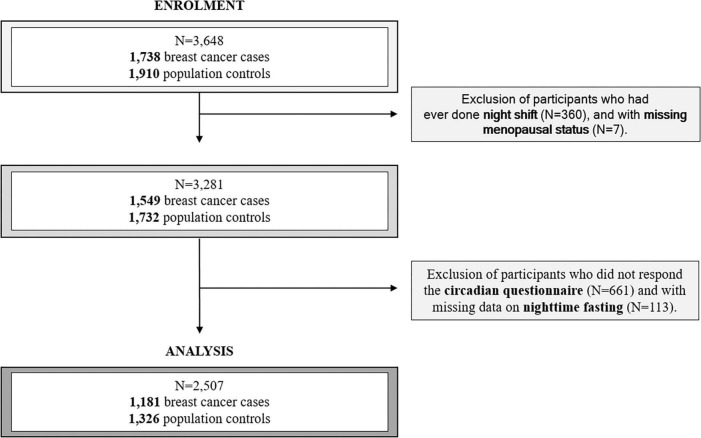
Participant flow diagram.

In this analysis, only breast cancer was examined. A total of 3,648 women were eligible for this analysis, including 1,738 breast cancer cases and 1,910 population controls. We excluded 360 women who reported ever working on the night shift and 7 with missing menopausal status (Figure 1). We excluded night shift workers, to focus our analysis mainly on the circadian disruption specifically related to nutritional behaviors and to avoid potential confounding with this other source of circadian disruption. We considered night shift work as working entirely or partly between 00:00 and 6:00 for 3 nights or more per month (9). We also excluded 661 women who did not respond to the circadian questionnaire and 113 who had missing information on nighttime fasting (Figure 1). Finally, 1,181 breast cancer cases and 1,326 population controls were included in these analyses.

### Data collection and variable assessment

Trained personnel administered an epidemiological questionnaire in a face-to-face interview. The questionnaire included information on socio-demographics, personal and family medical history, reproductive factors, medication, weight and height (corresponding to the year prior to study inclusion), recreational physical activity, and smoking (22). Using the Ainsworth classification (24), we assigned a physiological measure of energy expenditure (Metabolic Equivalent of Task, MET) to all recreational physical activities reported and we calculated the equivalent MET hour/week. We excluded data on physical activity corresponding to the 2 years before the interview to avoid any changes caused by the disease. We calculated body mass index (BMI) from self-reported weight and height. We also provided the participants with a previously validated food frequency questionnaire (FFQ) that was self-administered to evaluate nutritional behaviors over the previous year (22). The overall response rate was 88%. The questionnaire included an assessment of alcohol consumption between 30 and 40 years of age. Daily energy intake (kcal/day) and past daily consumption of ethanol (g/day) were estimated separately using the Centro de Enseñanza Superior de Nutrición y Dietética (CESNID) food composition table (25). As a proxy of a healthy diet, we also considered daily consumption of vegetables and fresh fruits (g/day).

Cases were classified into three subtypes based on pathology records, namely, (1) tumors with hormonal receptors either for estrogens or progesterone (labeled as positive hormonal receptors), (2) tumors with overexpression of the human epidermal growth factor 2 (HER2 +), and (3) tumors without hormonal receptors nor overexpression of HER2 (triple negative).

In total, 6 months to 5 years after enrollment in the study (median time 3 years), a telephonic interview was performed to assess circadian nutritional behaviors, timing of physical activity, and sleep patterns (questionnaire available on the study website; see text footnote 1). This interview also included a question on bedroom light during sleep assessed with a four-digit Likert scale (a) total darkness, (b) almost dark, (c) dim light, and (d) quite illuminated. Chronotype is the individual preference for the timing of circadian activity and has a genetic basis (26). This was also assessed in the circadian interview. Participants were asked to report their behaviors at mid-age (40 years of age) and the year before their inclusion in the study. Circadian nutritional behavior questions assessed the frequency of consumption of main meals and usual timing during weekdays and weekend days. We conducted the main analyses with behaviors at mid-age to avoid potential reverse causation from the more recent behaviors. We asked the participants about the frequency of breakfast consumption as never having breakfast, having breakfast only on weekends, having breakfast only on weekdays, and always having breakfast. Sleep duration was calculated as the difference between the time of turning off the lights and the time of awakening on weekdays and weekends. Nighttime fasting duration was calculated as the time elapsed between the last meal and breakfast the following day. For those participants that reported never having breakfast (1%) or having it only on weekends (< 1%), the time of lunch was considered as breakfast, understood as the broader concept of the time when the nightly fast was broken.

### Statistical analyses

We compared basic characteristics among cases and controls and in premenopausal and postmenopausal women separately.

To investigate the associations between nighttime fasting duration, time of breakfast, and breast cancer risk, we built logistic regression models and estimated odds ratios (ORs) and 95% confidence intervals (CIs). Models were adjusted for age (continuous, years), center (Madrid, Barcelona, Navarra, Gipuzkoa, Leon, Asturias, Huelva, Cantabria, Valencia, and Gerona), and educational level (less than primary school, primary school, secondary school, university). We also adjusted all models for well-established breast cancer risk factors: family history of breast cancer (no, yes), age at menarche (continuous, years), number of children (nulliparous, 1 or 2 children, 3 or more children), breastfeeding (parous women no breastfeeding, breastfeeding up to 6 months, breastfeeding 6–24 months, breastfeeding for more than 24 months, and nulliparous women), age at the first child (less than 20 years, from 20 to 35 years, more than 35 years, and nulliparous women), BMI 1 year before inclusion to the study (continuous, kg/m^2^), contraceptive use (never, ever), and hormonal replacement therapy (HRT) and menopausal status (premenopausal women, postmenopausal women who ever used HRT, and postmenopausal women who never used HRT). All the covariates included in the main model had less than 3% of missing values; therefore, we applied a complete case analysis. We initially explored separate models for the two exposures (nighttime fasting duration and time of breakfast) and then a model mutually adjusting both circadian nutritional behaviors.

There are well-established molecular and etiological differences between premenopausal and postmenopausal breast cancer (27), a pattern that has been replicated also for the effect of night shift work (28). We checked whether there was evidence of effect modification of the association between time of breakfast and nighttime fasting duration with breast cancer risk, by menopausal status by including an interaction term in the adjusted model and conducting a likelihood ratio test. We did follow the same procedure for chronotype and for HRT history (ever vs. never) among postmenopausal women.

We inspected the linearity of the association between nighttime fasting duration, time of breakfast, and breast cancer risk by building generalized additive models (GAMs). To test for linearity, we conducted an ANOVA comparing two models with the exposure of interest included with or without the smoothing term. None of the models showed a significant departure from linearity; therefore, we considered exposure variables as continuous. We further categorized nighttime fasting duration and time of breakfast according to the median point in controls: 11.00 h (interquartile range, IQR 10.00–12.00) and 8:00 a.m. (IQR 7:30–9:00 a.m.), respectively. We explored the correlation among both exposures and also with other circadian behaviors including those already examined in the previous MCC-Spain study on mistimed eating patterns (9).

Finally, in a multinomial logistic regression, we investigated the association of nighttime fasting duration and time of breakfast with the risk of breast cancer subtype, reporting relative risk (RR) ratios and examining differences between subtypes with the Wald test.

In sensitivity analyses, we explored adjustment for other lifestyle factors (daily alcohol intake, physical activity, daily caloric intake, and daily consumption of fruits and vegetables) and other potential risk factors for breast cancer including socioeconomic status, smoking, and age at menopause. We also explored further adjustment with other circadian behaviors including time of dinner, interval between dinner and sleep, indoor light-at-night, sleep duration, and chronotype. To investigate the potential influence of recall bias, we examined the association between nighttime fasting duration and time of breakfast with breast cancer risk using data reported for the year previous to diagnosis (or enrollment for controls) and checked the correlation with behaviors at mid-age. Finally, we explored the joint effects of nighttime fasting and breakfast timing in a model combining both exposures.

The statistical package R 4.0.5 was used to perform these analyses (R Foundation for Statistical Computing, Vienna, Austria).^2^

## Results

### Study population

The characteristics of our study population are shown in Table 1. The mean age of cases was 55 years (11.6 *SD*) and of controls 58 years (12.5 *SD*). Overall, cases were more likely to have a family history of breast cancer, to be premenopausal women, to have less children, and to have a higher past consumption of alcohol and daily energy. Sleep differed between controls and cases with a duration of 6.9 h (1.3 *SD*) and 7.1 h (1.3 *SD*), respectively. Only 9 controls (0.7%) reported never having breakfast, whereas 21 cases (1.8%) skipped breakfast. Moreover, the time of breakfast was later for cases (8.5, 1.4 *SD*) compared with controls (8.4, 1.4 *SD*). Nighttime fasting duration was similar between both groups (11.0, 1.6 *SD* controls and 11.1, 1.6 *SD* cases).

**TABLE 1:** Main characteristics of the study population.

	Controls (*N* = 1,326) mean (*SD*) or *N* (%)	Cases (*N* = 1,181) mean (*SD*) or *N* (%)
**Age (years)**	58.4 (12.5)	55.4 (11.6)
**BMI (kg/m^2^)**	25.7 (4.7)	25.9 (0.187)
**Education**		
Less than primary school	193 (14.6)	137 (11.6)
Primary school	412 (31.1)	403 (34.1)
Secondary school	438 (33.0)	407 (34.5)
University	283 (21.3)	234 (19.8)
**Score socioeconomic**		
Low	357 (27.8)	334 (28.3)
Medium	696 (54.2)	660 (55.9)
High	232 (18.1)	187 (15.8)
**Family history of breast cancer**		
Yes	124 (9.4)	175 (14.8)
No	1,202 (90.6)	1,006 (85.2)
**Diabetes**		
No	1,222 (92.4)	1,104 (93.9)
Yes	100 (7.6)	72 (6.1)
**Age at menarche (years)**	12.8 (1.6)	12.7 (1.5)
**Number of children**		
Nulliparous	236 (17.8)	243 (20.6)
1–2 children	745 (56.3)	694 (58.8)
3 children or more	342 (25.9)	243 (20.6)
**Age at first child**		
First child < 20 years old	52 (4.8)	46 (4.9)
First child 20–35 years old	957 (88.1)	811 (86.9)
Parous ≥ 35 years old	77 (7.1)	76 (8.1)
**Breastfeeding**		
Parous without breastfeeding	166 (15.3)	141 (15.5)
Parous breastfeeding for less than 6 months	294 (27.2)	269 (29.6)
Parous breastfeeding for 6–24 months	496 (45.8)	423 (46.5)
Parous breastfeeding for more than 24 months	126 (11.6)	76 (8.4)
**Contraceptive use**		
Never	648 (48.9)	603 (51.1)
Ever	677 (51.1)	577 (48.9)
**Menopausal status**		
Premenopausal	386 (29.1)	436 (36.9)
Postmenopausal	940 (70.9)	745 (63.1)
**Hormonal replacement therapy**		
Never	1,178 (92.0)	1,071 (92.5)
Ever	102 (8.0)	87 (7.5)
**Smoking**		
Never smoker	778 (58.7)	662 (56.1)
Past smoker	292 (22.0)	311 (26.4)
Current smoker	256 (19.3)	207 (17.5)
**Daily alcohol intake (g ethanol)**	6.0 (10.1)	6.9 (12.7)
**Daily caloric intake (Kcal)**	1,717.8 (537.4)	1,831.6 (610.5)
**Daily consumption of vegetables and fruits (g)**	557.0 (264.4)	555.8 (300.2)
**Physical activity^a^**		
Inactive	516 (38.9)	500 (42.3)
Poorly active	255 (19.2)	203 (17.2)
Moderately active	167 (12.6)	147 (12.4)
Very active	387 (29.2)	331 (28.0)
**Chronotype**		
Morning	508 (38.8)	426 (36.5)
Intermediate	528 (40.3)	464 (39.7)
Evening	273 (20.9)	278 (23.8)
Sleep duration (hours)	6.9 (1.3)	7.1 (1.3)
**Breakfast**		
Never	9 (0.7)	21 (1.8)
Only weekends	10 (0.8)	6 (0.5)
Only weekdays	20 (1.5)	24 (2.0)
Always	1,279 (97.0)	1,129 (95.7)
**Time of breakfast (start time, a.m.)**	8.4 (1.4)	8.5 (1.4)
**Nighttime fasting duration (hours)**	11.0 (1.6)	11.1 (1.6)

BMI, body mass index; N, sample size; SD, standard deviation.

^a^Physical activity was classified according to the annual mean of METS h/week. Inactive = 0 METS h/week; poorly active = 0.0001–8 METS h/week; moderately active = 8.0001–16 METS h/week; very active = more than 16.0001 METS h/week.

We found no correlation among controls between the time of breakfast and the time of last meal (Spearman’s correlation coefficient 0.09, Supplementary Figure 1) nor with the interval between dinner and time going to sleep (Spearman’s correlation coefficient 0.06). We found a high correlation between nighttime fasting duration and time of breakfast (Spearman’s correlation coefficient 0.8).

We explored characteristics of cases and controls by menopausal status (Supplementary Table 1). Postmenopausal cases tended to have a higher BMI compared with postmenopausal controls (27.1 vs. 26.3 kg/m^2^). Among premenopausal women, BMI was similar among both groups. Premenopausal cases had a longer nighttime fasting duration and a later breakfast compared with controls (11.0 vs. 10.6 and 8.6 vs. 8.2, respectively). In postmenopausal women, there were no differences in neither of these two nutritional circadian behaviors.

### Association of nighttime fasting duration and breast cancer risk

**FIGURE 2:**
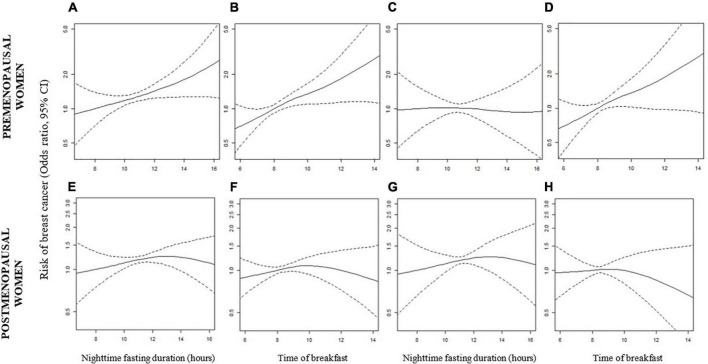
Generalized additive models showing the association between nighttime fasting duration, time of breakfast, and breast cancer risk in premenopausal **(A–D)** and postmenopausal women **(E–H)**. Models were adjusted for age, center, education, family history of breast cancer, menarche, number of children, BMI, contraceptive use, hormonal replacement therapy, breastfeeding, and age of the first child. In models **(C)**, **(D)**, **(G)**, and **(H)**, nighttime fasting duration and time of breakfast were mutually adjusted.

In all women, we observed no association between nighttime fasting duration and breast cancer risk after adjusting for the time of breakfast (Table 2, OR = 1.01, 95% CI: 0.93–1.11). For premenopausal women, we observed an association between nighttime fasting duration and breast cancer risk (OR = 1.11, 95% CI: 1.01–1.21), but no association was observed after adjusting for time of breakfast (OR = 0.99, 95% CI: 0.86–1.14). We observed the same tendency, in the GAMs (Figures 2A–C). Among postmenopausal women, we did not observe an association between nighttime fasting duration and breast cancer risk (Table 2, OR = 1.04, 95% CI: 0.93 – 1.17, model adjusted for time of breakfast). The absence of an association was also observed in Figures 2E,G. There was no significant evidence of effect modification by menopausal status.

**TABLE 2:** Logistic regression models investigating the association between nighttime fasting and time of breakfast with breast cancer risk.

All women				
	Controls *N* (%) or mean (*SD*)	Cases *N* (%) or mean (*SD*)	OR (95% CI)^a^	OR (95% CI)^b^
**Nighttime fasting**				
Continuous (hours)	11.0 (1.6)	11.1 (1.6)	1.05 (0.99–1.10)	1.01 (0.93–1.11)
≤11.00 h^c^	744 (60.4)	646 (57.9)	*Ref*	*Ref*
>11.00 h	488 (39.6)	470 (42.1)	1.12 (0.94–1.33)	1.02 (0.83–1.27)
**Time of breakfast**				
Continuous	8.4 (1.4)	8.5 (1.4)	1.06 (1.00–1.13)	1.05 (0.95–1.16)
≤8.00 a.m.^c^	648 (52.6)	518 (46.4)	*Ref*	*Ref*
>8.00 a.m.	584 (47.4)	598 (53.6)	1.27 (1.08–1.51)	1.25 (1.02–1.54)
**Premenopausal women**				
**Nighttime fasting**				
Continuous (hours)	10.6 (1.5)	11.0 (1.8)	1.11 (1.01–1.21)	0.99 (0.86–1.14)
≤11.00 h	265 (69.7)	262 (61.9)	*Ref*	*Ref*
>11.00 h	115 (30.3)	161 (38.1)	1.31 (0.96–1.78)	0.97 (0.66–1.43)
**Time of breakfast**				
Continuous	8.2 (1.3)	8.6 (1.6)	1.18 (1.06–1.31)	1.18 (1.01–1.40)
≤8.00 a.m.	227 (59.7)	205 (48.5)	*Ref*	*Ref*
>8.00 a.m.	153 (40.3)	218 (51.5)	1.53 (1.13–2.07)	1.40 (0.98–2.00)
**Postmenopausal women**				
**Nighttime fasting**				
Continuous (hours)	11.2 (1.7)	11.2 (1.5)	1.02 (0.95–1.09)	1.04 (0.93–1.17)
≤11.00 h	479 (56.2)	384 (55.4)	*Ref*	*Ref*
>11.00 h	373 (43.8)	309 (44.6)	1.06 (0.86–1.32)	1.07 (0.82–1.38)
**Time of breakfast**				
Continuous	8.5 (1.5)	8.5 (1.3)	1.01 (0.93–1.09)	0.97 (0.85–1.11)
≤8.00 a.m.	421 (49.4)	313 (45.2)	*Ref*	*Ref*
>8.00 a.m.	431 (50.6)	380 (54.8)	1.22 (0.98–1.52)	1.26 (0.97–1.62)

^a^Adjusted for age, center, education, family history of breast cancer, menarche, number of children, BMI, contraceptive use, hormonal replacement therapy, menopausal status, breastfeeding, and age of the first child.

^b^Same as a. Models for both exposures were mutually adjusted.

^c^Categorizations in both exposures were performed according to the median point among controls. N, sample size; OR, odds ratio; SD, standard deviation. The p-value for interaction between the time of breakfast and menopause = 0.021.

### Association of time of breakfast and breast cancer risk

In all women, having the first meal after 8 a.m. was associated with a 25% increase in the risk of having breast cancer compared with breakfast before 8 a.m. (Table 2, OR = 1.25, 95% CI: 1.02–1.54) after adjusting for nighttime fasting. In the continuous model, this association was weaker and non-significant (OR = 1.05, 95% CI: 0.95–1.16). This pattern was stronger for premenopausal women: the OR for having breakfast after 8 a.m. was 1.40 (95% CI: 0.98–2.00), while each hour later in the time of breakfast was associated with an 18% increase in the risk of having breast cancer (OR = 1.18, 95% CI: 1.01–1.40). Both models were adjusted for nighttime fasting duration. This association was linear (Figure 2D, *p*-value from ANOVA test = 0.35). In postmenopausal women, the pattern was less clear with a slightly increased risk observed in the categorical analysis but without a clear dose response (OR per hour later in breakfast = 0.97, 95% CI: 0.85–1.11) (Table 2). Figures 2F,H showed the same pattern. Finally, in a model combining our main exposures, we observed that an early breakfast (8 a.m. or before) was associated with a slightly reduced risk independently of the nighttime fasting duration (Supplementary Table 2). This was observed in all women and stratified by menopausal status.

We found a statistically significant effect modification of the association between the time of breakfast and breast cancer risk by menopausal status (*p*-value for interaction = 0.021). We found no effect modification by chronotype (*p*-value for interaction = 0.9) nor by HRT history (ever vs. never) among postmenopausal women (*p*-value for interaction = 0.5).

### Cancer subtype

In a multinomial logistic regression model, we explored the association of both nighttime fasting duration and time of breakfast with the RR of each breast cancer subtype. Among all women, we did not observe significant differences in nighttime fasting duration or time of breakfast and different cancer subtypes (Table 3). Among premenopausal women, later time of breakfast (per hour increase) was associated with a higher risk of HER2 + tumors compared with positive hormonal receptors and triple-negative (Table 3, RR = 1.38, 95% CI: 1.03–1.85, RR = 1.19, 95% CI: 0.99–1.42, and RR = 1.11, 95% CI: 0.69–1.78, *p*-value from Wald test = 0.03). No differences were observed for postmenopausal women by subtype.

**TABLE 3:** Multinomial logistic regression model investigating the association between nighttime fasting and time of breakfast with breast cancer risk subtype.

	Controls	HER2 +		+ Hormonal receptors		Triple-negative	
	Mean (*SD*) or *N* (%)	Mean (*SD*) or *N* (%)	RR (95% CI)^a^	Mean (*SD*) or *N* (%)	RR (95% CI)^a^	Mean (*SD*) or *N* (%)	RR (95% CI)^a^
**All women**							
Nighttime fasting (hours)	11.0 (1.6)	11.5 (1.6)	1.13 (0.96–1.34)	11.0 (1.6)	0.98 (0.89–1.08)	10.9 (1.6)	0.96 (0.75–1.22)
Time of breakfast	8.4 (1.4)	8.8 (1.6)	1.07 (0.89–1.28)	8.5 (1.3)	1.04 (0.93–1.17)	8.4 (1.4)	1.01 (0.75–1.35)
Total	1,232 (54.8)	200 (8.9)		740 (32.9)		75 (3.3)	
**Premenopausal women**							
Nighttime fasting (hours)	10.6 (1.5)	11.5 (1.8)	1.09 (0.84–1.41)	10.8 (1.7)	0.94 (0.81–1.10)	10.7 (1.6)	0.93 (0.63–1.37)
Time of breakfast	8.2 (1.3)	9.1 (1.8)	1.38 (1.03–1.85)	8.5 (1.4)	1.19 (0.99–1.42)	8.5 (1.5)	1.11 (0.69–1.78)
Total	380 (49.5)	71 (9.3)		290 (37.8)		26 (3.4)	
**Postmenopausal women**							
Nighttime fasting (hours)	11.2 (1.7)	11.4 (1.5)	1.19 (0.95–1.48)	11.2 (1.5)	1.02 (0.89–1.16)	11.0 (1.6)	1.02 (0.74–1.41)
Time of breakfast	8.5 (1.5)	8.7 (1.5)	0.92 (0.72–1.18)	8.5 (1.2)	0.98 (0.84–1.14)	8.4 (1.4)	0.91 (0.62–1.32)
Total	852 (57.6)	129 (8.7)		450 (30.4)		49 (3.3)	

^a^Models were adjusted for age, center, educational status, family history of breast cancer, menarche, number of children, BMI, contraceptive use, hormonal replacement therapy, menopausal status, breastfeeding, and age of the first child. Models were mutually adjusted for both exposures. Controls were considered as the reference group.

N, sample size; RR, relative Risk; SD, standard deviation.

### Sensitivity analyses

Adjustment for lifestyle factors and other potential breast cancer risk factors did not importantly change our estimates (Supplementary Tables 3, 4). We explored further adjustment of our models with other circadian behaviors (Supplementary Table 5). In all women, the additional adjustment of time of breakfast with the time of last meal (without nighttime fasting) strengthened the association between time of breakfast and breast cancer risk (OR = 1.05, 95% CI: 0.94–1.16 to 1.06 and 95% CI: 1.00–1.14). None of the other estimates in these models importantly changed after adjustment (Supplementary Table 5).

We also investigated the associations of interest using the behaviors reported as corresponding to the year prior to baseline. We observed associations between a later time of breakfast (per hour increase) and breast cancer in all, premenopausal and postmenopausal women (Supplementary Table 6, OR = 1.19, 95% CI: 1.08–1.31, OR = 1.21, 95% CI: 1.03–1.43, and OR = 1.18, 95% CI: 1.04–1.33, respectively). In none of the strata, nighttime fasting duration was associated with breast cancer risk after considering the time of breakfast (Supplementary Table 6).

We observed some differences in the correlation between data reported as corresponding to 40 years of age and to the previous year. For premenopausal women, the correlation was high for the time of breakfast (rho = 0.83), time of last meal (rho = 0.86), and nighttime fasting duration (rho = 0.82). For postmenopausal women, we found a low correlation for the time of breakfast (rho = 0.44), a moderate correlation for the time of last meal (rho = 0.52), and a low correlation for nighttime fasting duration (rho = 0.44).

## Discussion

This is one of the first epidemiological studies to investigate the association between circadian nutritional behaviors and breast cancer risk. We found that having a late breakfast was associated with an increased risk of breast cancer compared with an earlier breakfast. This pattern was stronger among premenopausal women and was observed across all chronotypes. We observed an association between nighttime fasting and breast cancer, especially among premenopausal women, which disappeared after adjusting for the time of breakfast.

There is few evidence available on circadian timing of diet and cancer risk. To the best of our knowledge, only one other epidemiological study examined the association between the time of breakfast and breast cancer risk (10). The results of this prospective study showed that each hour later in breakfast was associated with a 13% increase in the hazards of developing breast cancer risk (HR = 1.13, 95% 0.99–1.29, *p*-value 0.07) (10). In this French cohort, non-cases were younger (aged 45 years, 14.5 *SD*) compared with controls in this analysis (aged 58 years, 12.5 *SD*). Results were not stratified for menopausal status, which according to our results might be an effect modifier in this association. These and other factors such as the prospective study design in the NutriNet-Santé cohort or the exposure assessment could explain the differences between the results from the NutriNet-Santé study and the results presented in this study.

Two cross-sectional studies have suggested that prolonged nighttime fasting could reduce systemic inflammation and improve glycemic control, both potential breast cancer risk factors (12, 21). A prospective cohort study showed that elongating the nighttime fasting period could reduce breast cancer recurrence (11). In this study, the mean nighttime fasting duration was 12.5 (1.7, SD) h. Differences between our results and the results by Marinac et al. (11) could be also explained by the mean nighttime fasting of the study population. Although models were adjusted for a binary variable of eating after 8 PM, the time of breakfast was not considered. As suggested in our study and in a previous analysis from the MCC-Spain study, the time of breakfast was confounding the association between nighttime fasting duration and risk of cancer (29). In line with our results, in the NutriNet-Santé study nighttime fasting duration was not associated with breast cancer after adjusting for the time of the first meal. In the French cohort, the mean nighttime fasting duration was 11.9 (1.2, *SD*).

Several hypotheses could explain the association between the time of breakfast and breast cancer risk. Breakfast skipping might be compensated with a higher intake later on the day (6). This could be also the case for a later time of breakfast, but even after adjusting for daily caloric intake, no changes were observed. Similarly, regular breakfast consumption has been linked with healthier lifestyle behaviors (6). It could be that the observations for early breakfast are also indicative of a “healthy user bias.” We explored adjustment for alcohol intake, vegetable and fruit intake, and physical activity, and we did not observe important changes.

The association is biologically plausible. Delaying breakfast could be associated with worse glycemic control (15), lipid profile (17), inflammation (16), and alterations in the cortisol rhythm (30), which may then lead to breast cancer risk (11, 12, 31, 32). In animal models, it has been shown that skipping the analogous breakfast, delaying the first active-phase meal by 4 h, can be associated with increased visceral fat (33), increased hepatic lipid accumulation (34), and with a phase delay in the expression of circadian genes in the liver and fat tissue (35, 36). A randomized clinical trial showed that skipping breakfast acutely altered the regulation of clock and clock-controlled genes (37). Supporting this hypothesis, the downregulation of clock genes has been correlated with breast cancer (38).

The differences in menopausal status could be explained by an increased susceptibility of breast tissue to circadian disruption in earlier life stages (28). It could also be that the potential for recall bias is greater among postmenopausal women (older women) with a long time elapsed since exposure. In fact, the correlation between mid-age behaviors and the previous year to the inclusion in this study was lower in postmenopausal women. The strongest association between the time of breakfast and breast cancer risk in premenopausal women was observed in HER2 + cases. However, there is less evidence on differences in circadian parameters and breast cancer subtypes (3). Further studies are needed to confirm and understand these differences.

The main strengths of this study are the large sample size of the study population, which enabled the stratification of our results by menopausal status, and the detailed information on circadian nutritional behaviors. This investigation gives new insights and future questions on the impact of chrononutrition in cancer, an emerging field of study that deserves more attention. The main limitation of this study is the potential for recall bias since women were asked for behaviors at 40 years of age. Circadian nutritional behaviors were assessed at one single time point, which may affect the validity of these exposures. Finally, given the observational nature of the study and its design, residual confounding cannot be completely ruled out, and causal interpretation of these findings should be taken with caution.

Our results suggest that delaying circadian nutritional behaviors, specifically having a late breakfast, is associated with increased breast cancer risk, especially among premenopausal women. Together with our previous study on other circadian timing aspects, this study suggests that when to eat may also be an important aspect of healthy nutritional behaviors, influencing cancer risk. If these results are confirmed by prospective studies and clinical trials, public nutritional recommendations may consider including timing aspects aside from the quality and quantity components of the diet.

## Data availability statement

The raw data supporting the conclusions of this article will be made available by the authors, without undue reservation.

## Ethics statement

The studies involving human participants were reviewed and approved by CEIC 2008/3123/I. The patients/participants provided their written informed consent to participate in this study.

## Author contributions

BP-G, EA, TF, PA, IG-A, VM, JA, GF-T, AM-B, RM-G, NA, GC-V, MG, AM, MP, and MK performed the data acquisition. AP-C and AE performed the data curation. MP and MK carried funding acquisition. DR and MK performed the supervision of the study. AP-C wrote the first draft of the manuscript. All authors contributed to the study conception and design, commented on previous versions of the manuscript and read and approved the final manuscript.

## Footnotes

1
http://www.mccspain.org

2
http://www.R-project.org/
