# Supplementary material for: Association of time of breakfast and nighttime fasting duration with breast cancer risk in the multicase-control study in Spain
Source: Front Nutr. 2022 Aug 11;9:941477. doi: 10.3389/fnut.2022.941477 (PMC9404378; doi:10.3389/fnut.2022.941477)
Supplement: Supplementary file 1 [file Data_Sheet_1.docx]

Supplementary Material

**Supplementary figure 1.** Heat map of correlations among circadian behaviors. Values in the figure correspond to Spearman’s correlation coefficient.


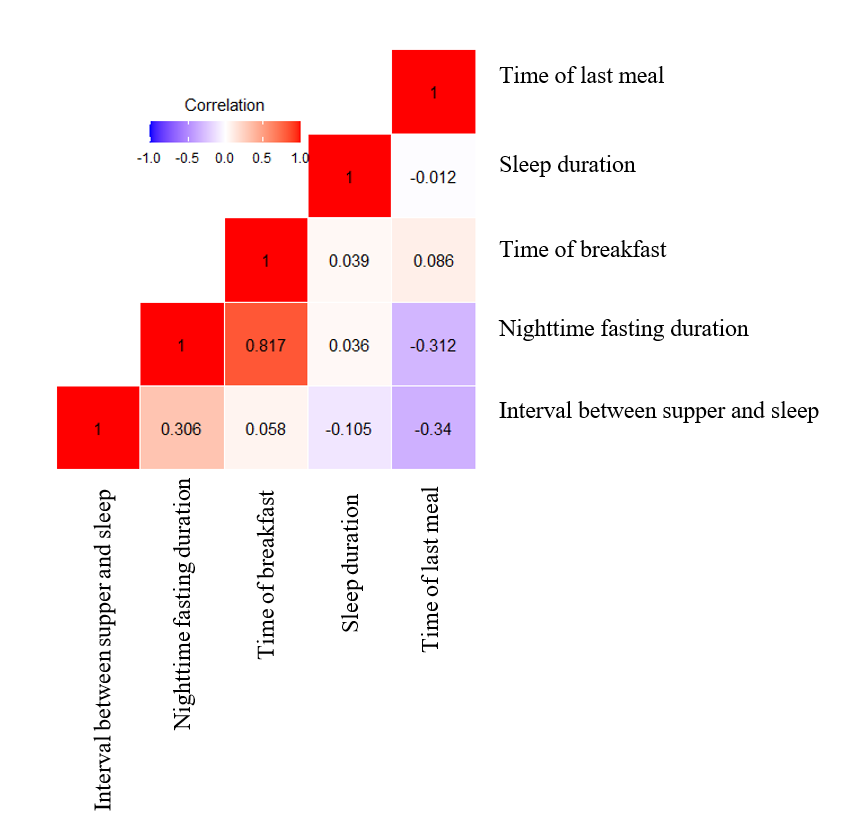


| Supplementary table 1. Baseline characteristics of the study population by menopausal status. | | | | |
| --- | --- | --- | --- | --- |
| Variable | **Premenopausal women** | | **Postmenopausal women** | |
|  | **Controls**  **(N = 386)**  **Mean (SD) or N (%)** | **Cases**  **(N = 436)**  **Mean (SD) or N (%)** | **Controls**  **(N = 940)**  **Mean (SD) or N (%)** | **Cases**  **(N = 745)**  **Mean (SD) or N (%)** |
| Age (years) | 43.9 (5.9) | 44.3 (6.3) | 64.3 (9.2) | 61.9 (8.7) |
| BMI (kg/m^2^) | 24.2 (4.3) | 24.0 (3.7) | 26.3 (4.7) | 27.1 (5.0) |
| Education |  |  |  |  |
| Less than primary school | 10 (2.6) | 10 (2.3) | 183 (19.5) | 127 (17.0) |
| Primary school | 66 (17.1) | 92 (21.1) | 346 (36.8) | 311 (41.7) |
| Secondary school | 163 (42.2) | 198 (45.4) | 275 (29.3) | 209 (28.1) |
| University | 147 (38.1) | 136 (31.2) | 136 (14.5) | 98 (13.2) |
| Score socioeconomic |  |  |  |  |
| Low | 46 (11.9) | 60 (13.8) | 311 (34.6) | 274 (36.8) |
| Medium | 221 (57.3) | 270 (61.9) | 475 (52.8) | 390 (52.3) |
| High | 119 (30.8) | 106 (24.3) | 113 (12.6) | 81 (10.9) |
| Family history of breast cancer |  |  |  |  |
| Yes | 21 (5.4) | 63 (14.4) | 103 (11.0) | 112 (15.0) |
| No | 365 (94.6) | 373 (85.6) | 837 (89.0) | 633 (85.0) |
| Diabetes |  |  |  |  |
| Yes | 8 (2.1) | 6 (1.4) | 92 (9.8) | 66 (8.9) |
| No | 376 (97.9) | 428 (98.6) | 846 (90.2) | 676 (91.1) |
| Age at menarche (years) | 12.7 (1.4) | 12.6 (1.5) | 12.9 (1.6) | 12.8 (1.6) |
| Number of children |  |  |  |  |
| Nulliparous | 97 (25.3) | 110 (25.2) | 139 (14.8) | 133 (17.9) |
| 1-2 children | 251 (65.5) | 287 (65.8) | 494 (52.6) | 407 (54.7) |
| 3 children or more | 35 (9.1) | 39 (8.9) | 307 (32.7) | 204 (27.4) |
| Age at first child |  |  |  |  |
| First child < 20 years old | 22 (7.7) | 20 (6.2) | 30 (3.8) | 26 (4.3) |
| First child 20 to 35 years old | 220 (76.9) | 257 (79.1) | 737 (92.1) | 554 (91.1) |
| Parous ≥35 years old | 44 (15.4) | 48 (14.8)) | 33 (4.1) | 28 (4.6) |
| Breastfeeding |  |  |  |  |
| Parous without breastfeeding | 40 (14.0) | 46 (14.5) | 126 (15.8) | 95 (16.1) |
| Parous breastfeeding for less than 6 months | 92 (32.3) | 101 (31.8) | 202 (25.3) | 168 (28.4) |
| Parous breastfeeding for 6 to 24 months | 122 (42.8) | 148 (46.5) | 374 (46.9) | 275 (46.5) |
| Parous breastfeeding for more than 24 months | 31 (10.9) | 23 (7.2) | 95 (11.9) | 53 (9.0) |
| Contraceptive use |  |  |  |  |
| Never | 111 (28.8) | 153 (35.2) | 537 (57.1) | 450 (60.4) |
| Ever | 274 (71.2) | 282 (64.8) | 403 (42.9) | 295 (39.6) |
| Hormonal replacement therapy |  |  |  |  |
| Never | NA | NA | 793 (88.6) | 638 (88.1) |
| Ever | NA | NA | 102 (11.4) | 86 (11.9) |
| Smoking |  |  |  |  |
| Never smoker | 162 (42.0) | 177 (40.6) | 616 (65.5) | 485 (65.2) |
| Past smoker | 108 (28.0) | 155 (35.6) | 184 (19.6) | 156 (21.0) |
| Current smoker | 116 (30.1) | 104 (23.9) | 140 (14.9) | 103 (13.8) |
| Alcohol intake (g ethanol) | 5.6 (8.7) | 6.1 (10.8) | 6.1 (10.5) | 7.4 (13.7) |
| Daily caloric intake (Kcal) | 1743.5 (554.7) | 1913.9 (667.3) | 1707.4 (530.2) | 1771.9 (566.2) |
| Daily consumption of vegetables and fruits (g) | 479.3 (252.1) | 503.2 (293.4) | 588.4 (262.9) | 587.0 (300.1) |
| Physical activity ^a^ |  |  |  |  |
| Inactive | 153 (39.6) | 297 (45.2) | 363 (38.7) | 303 (40.7) |
| Poorly active | 95 (24.6) | 92 (21.1) | 160 (17.0) | 111 (14.9) |
| Moderately active | 49 (12.7) | 55 (12.6) | 118 (12.6) | 92 (12.3) |
| Very active | 89 (23.1) | 92 (21.1) | 298 (31.7) | 239 (32.1) |
| Chronotype |  |  |  |  |
| Morning | 134 (34.7) | 154 (35.6) | 374 (40.5) | 272 (37.0) |
| Intermediate | 166 (43.0) | 172 (39.8) | 362 (39.2) | 292 (39.7) |
| Evening | 86 (22.3) | 106 (24.5) | 187 (20.3) | 172 (23.4) |
| Sleep duration (hours) | 7.1 (1.2) | 7.2 (1.0) | 6.9 (1.3) | 7.0 (1.4) |
| Breakfast |  |  |  |  |
| Never | 2 (0.5) | 9 (2.1) | 7 (0.8) | 12 (1.6) |
| Only weekends | 1 (0.3) | 4 (0.9) | 9 (1.0) | 2 (0.3) |
| Only weekdays | 6 (1.6) | 7 (1.6) | 14 (1.5) | 17 (2.3) |
| Always | 376 (97.7) | 415 (95.4) | 903 (96.8) | 714 (95.8) |
| Time of breakfast | 8.2 (1.3) | 8.6 (1.6) | 8.5 (1.4) | 8.5 (1.3) |
| Nighttime fasting duration (hours) | 10.6 (1.5) | 11.0 (1.7) | 11.2 (1.6) | 11.2 (1.5) |

^a^ Physical activity was classified according to the annual mean of METS h / week. Inactive = 0 METS h/week; Poorly active = 0.0001 to 8 METS h/week; Moderately active = 8.0001 to 16 METS h/week; Very active = More than 16.0001 METS h/week. BMI= Body mass index; N= Sample size; OR= odds ratio; SD=Standard deviation.

| Supplementary table 2. Logistic regression models investigating the association between nighttime fasting and time of breakfast with breast cancer risk in a combined exposure variable. | | | |
| --- | --- | --- | --- |
| ALL WOMEN | | | |
|  | **Controls N (%)** | **Cases**  **N (%)** | **OR (95% CI) ^a^** |
| Short nighttime fasting and late breakfast ^b^ | 176 (13.3) | 191 (16.2) | *Ref* |
| Short nighttime fasting and early breakfast | 603 (45.5) | 483 (40.9) | 0.78 (0.61 – 1.00) |
| Long nighttime fasting and late breakfast | 467 (35.2) | 441 (37.3) | 0.97 (0.75 – 1.26) |
| Long nighttime fasting and early breakfast | 80 (6.0) | 66 (5.6) | 0.72 (0.47 – 1.09) |
| PREMENOPAUSAL WOMEN | | | |
|  | **Controls N (%)** | **Cases**  **N (%)** | **OR (95% CI) ^a^** |
| Short nighttime fasting and late breakfast | 56 (12.8) | 75 (15.6) | *Ref* |
| Short nighttime fasting and early breakfast | 213 (41.5) | 189 (39.5) | 0.66 (0.43 – 1.01) |
| Long nighttime fasting and late breakfast | 99 (39.1) | 154 (38.5) | 1.03 (0.65 – 1.61) |
| Long nighttime fasting and early breakfast | 18 (4.7) | 18 (4.1) | 0.68 (0.31 – 1.50) |
| POSTMENOPAUSAL WOMEN | | | |
|  | **Controls N (%)** | **Cases**  **N (%)** | **OR (95% CI) ^a^** |
| Short nighttime fasting and late breakfast | 120 (12.8) | 116 (15.6) | *Ref* |
| Short nighttime fasting and early breakfast | 390 (41.5) | 294 (39.5) | 0.78 (0.57 – 1.08) |
| Long nighttime fasting and late breakfast | 368 (39.1) | 287 (38.5) | 0.93 (0.67 – 1.28) |
| Long nighttime fasting and early breakfast | 62 (6.6) | 48 (6.4) | 0.72 (0.43 – 1.19) |
| ^a^ Adjusted for age, center, education, family history of breast cancer, menarche, number of children, BMI, contraceptive use, hormonal replacement therapy and menopausal status, breastfeeding and age at first child. ^b^ Nighttime fasting: short interval ≤ 11 hr.; long interval >11hr. Time of breakfast: Early breakfast ≤8:00 AM; Late breakfast >8:00AM. | | | |

| Supplementary table 3. Logistic regression models exploring further adjustment of the association between nighttime fasting and time of breakfast with breast cancer risk for other lifestyle factors. Complete case analysis including subjects with information on all covariates. | | | | | | | |
| --- | --- | --- | --- | --- | --- | --- | --- |
| ALL WOMEN | | | | | | | |
|  | **Controls (N=1107) mean (SD)** | **Cases (N=993) mean (SD)** | **OR (95% CI) ^a^** | **OR (95% CI) ^b^** | **OR (95% CI) ^c^** | **OR (95% CI) ^d^** | **OR (95% CI) ^e^** |
| Nighttime fasting (hours) | 11.0 (1.6) | 11.0 (1.6) | 1.01 (0.92-1.11) | 1.02 (0.93-1.12) | 1.02 (0.92-1.12) | 1.02 (0.93-1.12) | 1.01 (0.92-1.11) |
| Time of breakfast | 8.4 (1.4) | 8.5 (1.4) | 1.05 (0.94-1.17) | 1.04 (0.93-1.16) | 1.04 (0.93-1.16) | 1.04 (0.93-1.16) | 1.05 (0.94-1.17) |
| PREMENOPAUSAL WOMEN | | | | | | | |
|  | **Controls (N=337) mean (SD)** | **Cases (N=381) mean (SD)** | **OR (95% CI) ^a^** | **OR (95% CI) ^b^** | **OR (95% CI) ^c^** | **OR (95% CI) ^d^** | **OR (95% CI) ^e^** |
| Nighttime fasting (hours) | 10.6 (1.5) | 10.9 (1.7) | 0.98 (0.84-1.13) | 0.98 (0.85-1.14) | 0.98 (0.85-1.14) | 0.98 (0.85-1.14) | 0.97 (0.84-1.12) |
| Time of breakfast | 8.2 (1.2) | 8.5 (1.5) | 1.19 (1.00-1.42) | 1.19 (1.00-1.42) | 1.18 (0.99-1.41) | 1.17 (0.98-1.41) | 1.21 (1.01-1.44) |
| POSTMENOPAUSAL WOMEN | | | | | | | |
|  | **Controls (N=770) mean (SD)** | **Cases (N=612) mean (SD)** | **OR (95% CI) ^a^** | **OR (95% CI) ^b^** | **OR (95% CI) ^c^** | **OR (95% CI) ^d^** | **OR (95% CI) ^e^** |
| Nighttime fasting (hours) | 11.2 (1.7) | 11.2 (1.5) | 1.04 (0.92-1.18) | 1.05 (0.93-1.19) | 1.05 (0.93-1.19) | 1.05 (0.93-1.19) | 1.05 (0.92-1.19) |
| Time of breakfast | 8.5 (1.5) | 8.5 (1.3) | 0.97 (0.84-1.12) | 0.96 (0.83-1.11) | 0.96 (0.83-1.11) | 0.96 (0.83-1.11) | 0.97 (0.84-1.12) |
| ^a^ Basic model. Adjusted for age, center, education, family history of breast cancer, menarche, number of children, BMI, contraceptive use, hormonal replacement therapy and menopausal status, breastfeeding and age at first child. Both exposures were mutually adjusted.  ^b^ Basic model adjusted for daily alcohol intake.  ^c^ Basic model adjusted for physical activity.  ^d^ Basic model adjusted for daily caloric intake.  ^e^ Basic model adjusted for daily consumption of fruits and vegetables.  N= Sample size; OR= odds ratio; SD= Standard deviation. | | | | | | | |

| Supplementary table 4. Logistic regression models exploring further adjustment of the association between nighttime fasting and time of breakfast with breast cancer risk for potential breast cancer risk factors. Complete cases analysis including subjects with information on all covariates included in the models. | | | | | | | |
| --- | --- | --- | --- | --- | --- | --- | --- |
| ALL WOMEN | | | | | | | |
|  | **Controls (N=1143) mean (SD)** | **Cases (N=1032)**  **mean (SD)** | **OR (95% CI) ^a^** | **OR (95% CI) ^b^** | | **OR (95% CI) ^c^** | **OR (95% CI) ^d^** |
| Nighttime fasting (hours) | 11.0 (1.6) | 11.1 (1.6) | 1.02 (0.93-1.12) | 1.02 (0.93-1.12) | | 1.02 (0.93-1.11) | 1.02 (0.93-1.12) |
| Time of breakfast | 8.4 (1.4) | 8.6 (1.4) | 1.04 (0.94-1.16) | 1.04 (0.94-1.16) | | 1.05 (0.95-1.17) | 1.04 (0.94-1.16) |
| PREMENOPAUSAL WOMEN | | | | | | | |
|  | **Controls (N=380) mean (SD)** | **Cases (N=423) mean (SD)** | **OR (95% CI) ^a^** | | **OR (95% CI) ^b^** | **OR (95% CI) ^c^** | **OR (95% CI) ^d^** |
| Nighttime fasting (hours) | 10.6 (1.5) | 11.0 (1.8) | 0.99 (0.86-1.14) | | 0.99 (0.86-1.14) | 0.98 (0.85-1.13) | NA |
| Time of breakfast | 8.2 (1.3) | 8.6 (16) | 1.18 (1.01-1.40) | | 1.18 (1.01-1.40) | 1.21 (1.03-1.44) | NA |
| POSTMENOPAUSAL WOMEN | | | | | | | |
|  | **Controls (N=763) mean (SD)** | **Cases (N=609) mean (SD)** | **OR (95% CI) ^a^** | | **OR (95% CI) ^b^** | **OR (95% CI) ^c^** | **OR (95% CI) ^d^** |
| Nighttime fasting (hours) | 11.2 (1.6) | 11.2 (1.5) | 1.06 (0.94-1.20) | | 1.06 (0.94-1.21) | 1.06 (0.93-1.20) | 1.06 (0.94-1.21) |
| Time of breakfast | 8.5 (1.5) | 8.5 (1.3) | 0.96 (0.83-1.11) | | 0.96 (0.83-1.10) | 0.97 (0.84-1.11) | 0.96 (0.83-1.11) |
| ^a^ Basic model. Adjusted for age, center, education, family history of breast cancer, menarche, number of children, BMI, contraceptive use, hormonal replacement therapy and menopausal status, breastfeeding and age at first child. Both exposures were mutually adjusted.  ^b^ Basic model adjusted for score socioeconomic.  ^c^ Basic model adjusted for smoking.  ^d^ Basic model adjusted for age at menopause.  NA=Not applicable; N= Sample size; OR= odds ratio; SD= Standard deviation. | | | | | | | |

| Supplementary table 5. Logistic regression models exploring further adjustment of the association between nighttime fasting and time of breakfast with breast cancer risk for other circadian behaviors. Complete cases analysis including subjects with information on all covariates included in the models. | | | | | | | | |
| --- | --- | --- | --- | --- | --- | --- | --- | --- |
| ALL WOMEN | | | | | | | | |
|  | **Controls (N=1128) mean (SD)** | **Cases (N=1072) mean (SD)** | **OR (95% CI) ^a^** | **OR (95% CI) ^b^** | **OR (95% CI) ^c^** | **OR (95% CI) ^d^** | **OR (95% CI) ^e^** | **OR (95% CI) ^f^** |
| Nighttime fasting (hours) | 11.0 (1.6) | 11.1 (1.6) | 1.02 (0.93-1.11) | 1.05 (0.99-1.11) | 1.03 (0.94-1.14) | 1.02 (0.93-1.12) | 1.01 (0.93-1.11) | 1.02 (0.93-1.12) |
| Time of breakfast | 8.4 (1.4) | 8.5 (1.4) | 1.05 (0.94-1.16) | 1.06 (1.00-1.14) | 1.04 (0.93-1.15) | 1.04 (0.94-1.16) | 1.05 (0.94-1.16) | 1.04 (0.93-1.16) |
| PREMENOPAUSAL WOMEN | | | | | | | | |
|  | **Controls (N=363) mean (SD)** | **Cases (N=407) mean (SD)** | **OR (95% CI) ^a^** | **OR (95% CI) ^b^** | **OR (95% CI) ^c^** | **OR (95% CI) ^d^** | **OR (95% CI) ^e^** | **OR (95% CI) ^f^** |
| Nighttime fasting (hours) | 10.6 (1.6) | 10.9 (1.7) | 0.98 (0.85-1.13) | 1.09 (0.99-1.20) | 0.99 (0.86-1.14) | 0.98 (0.85-1.13) | 0.97 (0.84-1.12) | 0.97 (0.84-1.12) |
| Time of breakfast | 8.2 (1.3) | 8.6 (1.5) | 1.18 (1.00-1.40) | 1.16 (1.04-1.30) | 1.19 (1.00-1.41) | 1.17 (0.99-1.38) | 1.19 (1.01-1.41) | 1.18 (1.00-1.41) |
| POSTMENOPAUSAL WOMEN | | | | | | | | |
|  | **Controls (N=775) mean (SD)** | **Cases (N=672) mean (SD)** | **OR (95% CI) ^a^** | **OR (95% CI) ^b^** | **OR (95% CI) ^c^** | **OR (95% CI) ^d^** | **OR (95% CI) ^e^** | **OR (95% CI) ^f^** |
| Nighttime fasting (hours) | 11.1 (1.6) | 11.2 (1.5) | 1.06 (0.94-1.19) | 1.03 (0.96-1.11) | 1.07 (0.95-1.22) | 1.05 (0.93-1.19) | 1.05 (0.93-1.19) | 1.07 (0.94-1.21) |
| Time of breakfast | 8.5 (1.4) | 8.5 (1.3) | 0.96 (0.83-1.10) | 1.01 (0.93-1.10) | 0.95 (0.82-1.09) | 0.96 (0.84-1.10) | 0.96 (0.93-1.10) | 0.95 (0.83-1.10) |
| ^a^ Basic model. Adjusted for age, center, education, family history of breast cancer, menarche, number of children, BMI, contraceptive use, hormonal replacement therapy and menopausal status, breastfeeding and age at first child. Both exposures were mutually adjusted.  ^b^ Basic model adjusted for time of last meal. In this case, both exposures were not mutually adjusted to avoid over adjustment of circadian variables.  ^c^ Basic model adjusted for interval between dinner and sleep.  ^d^ Basic model adjusted for indoor ALAN.  ^e^ Basic model adjusted for sleep duration.  ^f^ Basic model adjusted for chronotype.  N= Sample size; OR= odds ratio; SD= Standard deviation. | | | | | | | | |

| Supplementary table 6. Logistic regression models of the association between nighttime fasting and time of breakfast with breast cancer risk using data corresponding to behaviors the year previous to the inclusion in the study. | | | |
| --- | --- | --- | --- |
| ALL WOMEN | | | |
|  | **Controls (N=1266) mean (SD)** | **Cases (N=1130) mean (SD)** | **OR (95% CI) ^a^** |
| Nighttime fasting (hours) | 11.2 (1.5) | 11.3 (1.7) | 0.96 (0.89-1.04) |
| Time of breakfast | 8.7 (1.2) | 8.9 (1.4) | 1.19 (1.08-1.31) |
| PREMENOPAUSAL WOMEN | | | |
|  | **Controls (N=381) mean (SD)** | **Cases (N=423) mean (SD)** | **OR (95% CI) ^a^** |
| Nighttime fasting (hours) | 10.6 (1.5) | 11.0 (1.8) | 1.01 (0.88-1.16) |
| Time of breakfast | 8.3 (1.3) | 8.7 (1.5) | 1.21 (1.03-1.43) |
| POSTMENOPAUSAL WOMEN | | | |
|  | **Controls (N=885) mean (SD)** | **Cases (N=707) mean (SD)** | **OR (95% CI) ^a^** |
| Nighttime fasting (hours) | 11.5 (1.5) | 11.5 (1.6) | 0.95 (0.87-1.05) |
| Time of breakfast | 8.9 (1.2) | 9.0 (1.2) | 1.18 (1.04-1.33) |
| ^a^ Adjusted for age, center, education, family history of breast cancer, menarche, number of children, BMI, contraceptive use, hormonal replacement therapy and menopausal status, breastfeeding and age at first child. Both exposures were mutually adjusted.  N= Sample size; OR= odds ratio; SD= Standard deviation. | | | |
